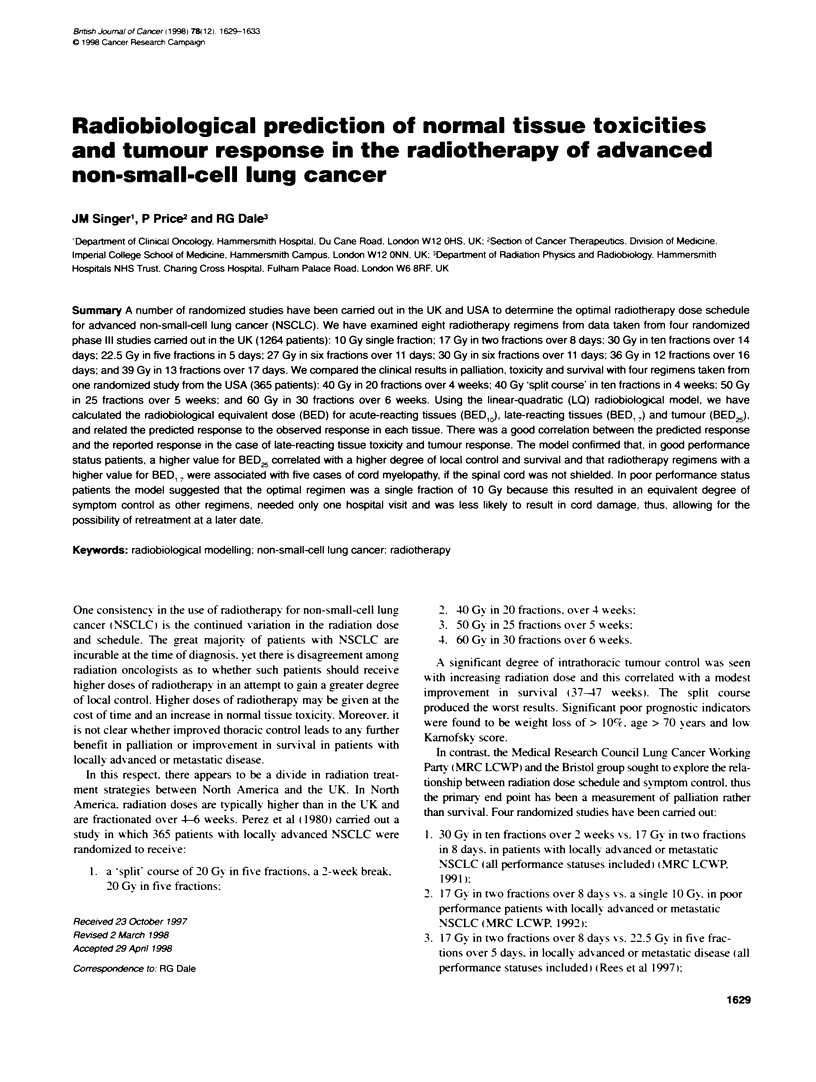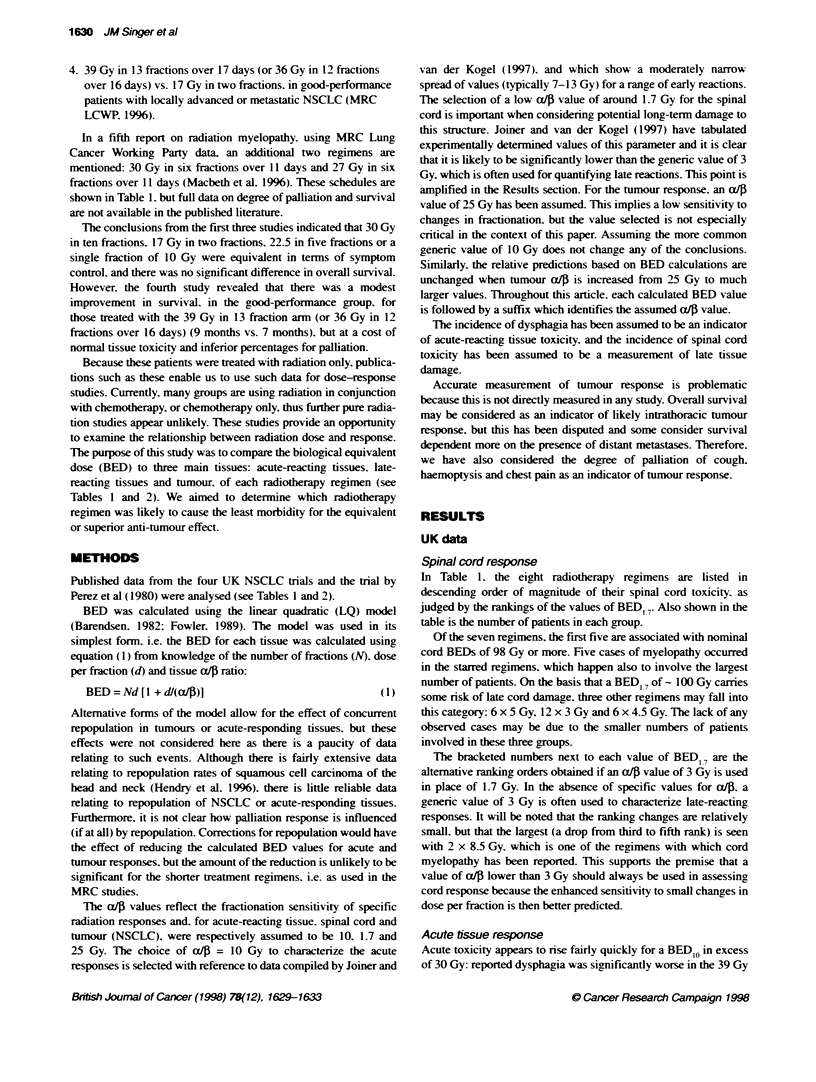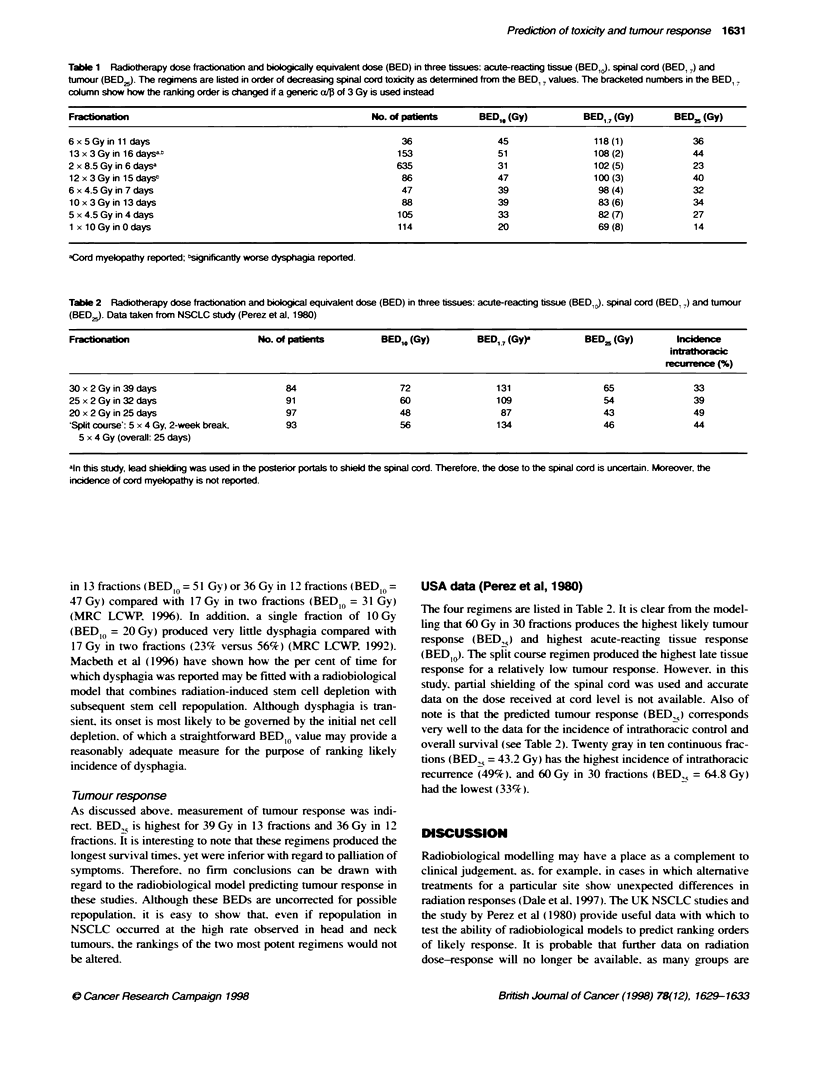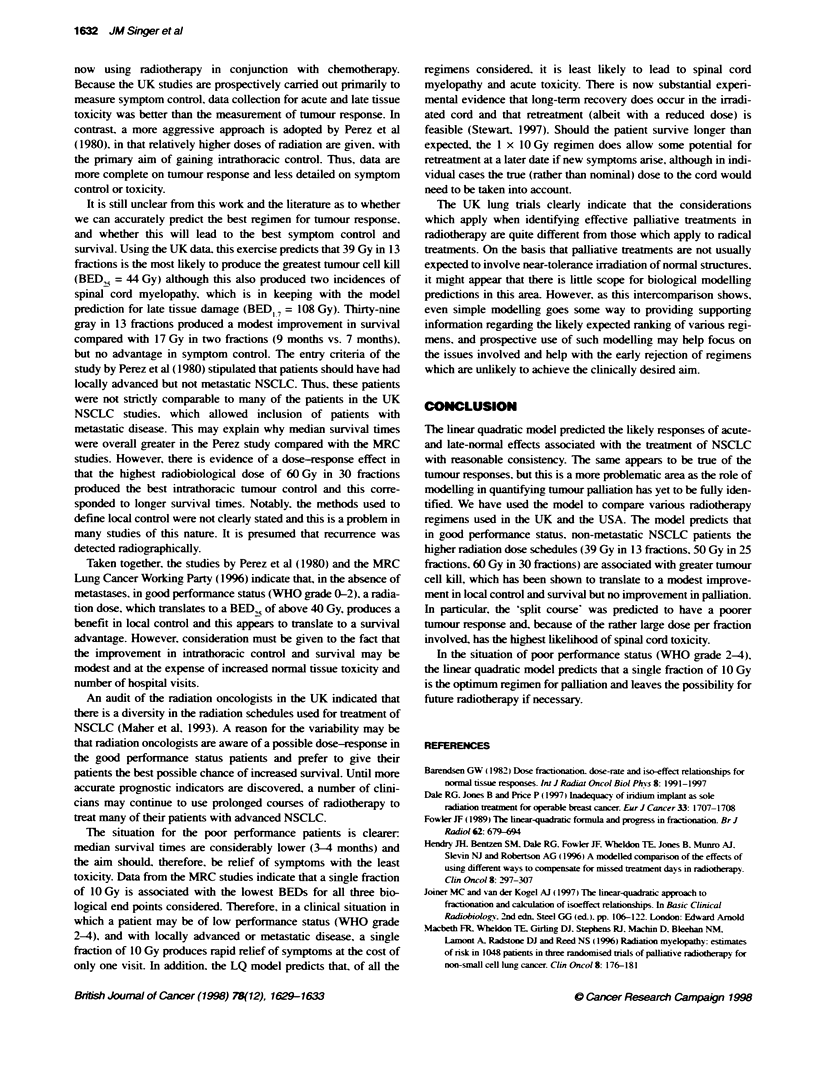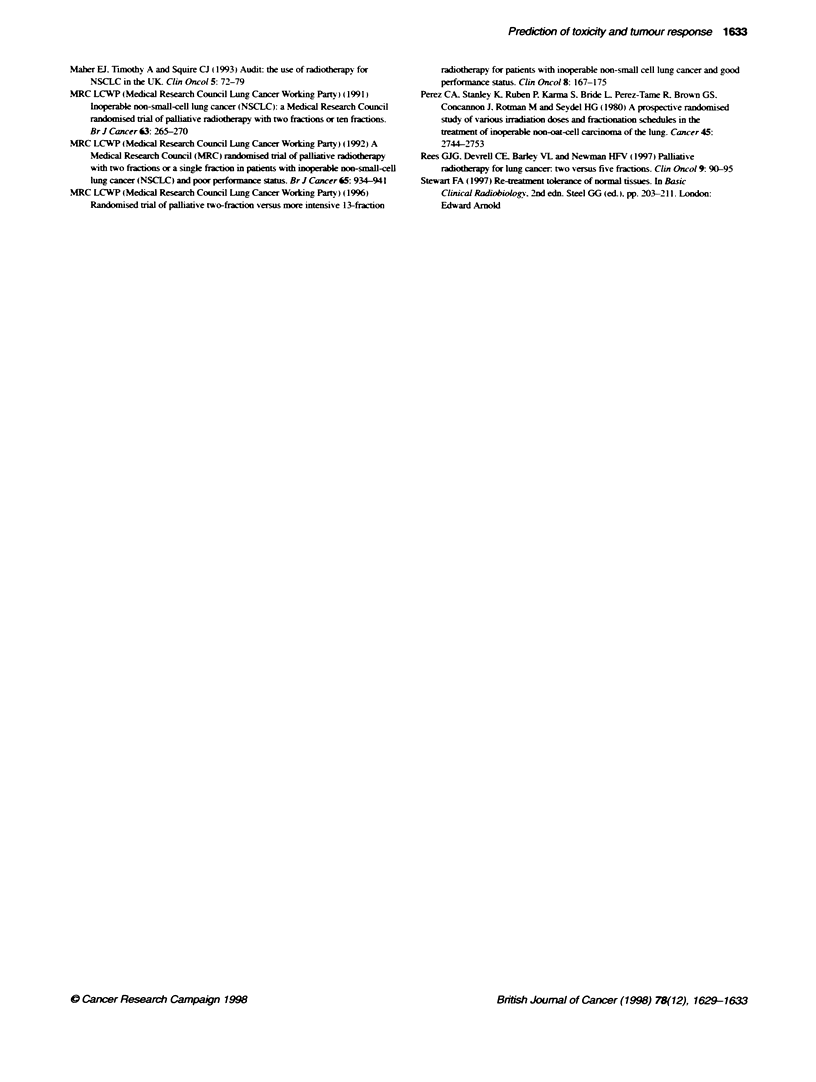# Radiobiological prediction of normal tissue toxicities and tumour response in the radiotherapy of advanced non-small-cell lung cancer.

**DOI:** 10.1038/bjc.1998.734

**Published:** 1998-12

**Authors:** J. M. Singer, P. Price, R. G. Dale

**Affiliations:** Department of Clinical Oncology, Hammersmith Hospital, London, UK.

## Abstract

A number of randomized studies have been carried out in the UK and USA to determine the optimal radiotherapy dose schedule for advanced non-small-cell lung cancer (NSCLC). We have examined eight radiotherapy regimens from data taken from four randomized phase III studies carried out in the UK (1264 patients): 10 Gy single fraction; 17 Gy in two fractions over 8 days; 30 Gy in ten fractions over 14 days; 22.5 Gy in five fractions in 5 days; 27 Gy in six fractions over 11 days; 30 Gy in six fractions over 11 days; 36 Gy in 12 fractions over 16 days; and 39 Gy in 13 fractions over 17 days. We compared the clinical results in palliation, toxicity and survival with four regimens taken from one randomized study from the USA (365 patients): 40 Gy in 20 fractions over 4 weeks; 40 Gy 'split course' in ten fractions in 4 weeks; 50 Gy in 25 fractions over 5 weeks; and 60 Gy in 30 fractions over 6 weeks. Using the linear-quadratic (LQ) radiobiological model, we have calculated the radiobiological equivalent dose (BED) for acute-reacting tissues (BED10), late-reacting tissues (BED1.7) and tumour (BED25), and related the predicted response to the observed response in each tissue. There was a good correlation between the predicted response and the reported response in the case of late-reacting tissue toxicity and tumour response. The model confirmed that, in good performance status patients, a higher value for BED25 correlated with a higher degree of local control and survival and that radiotherapy regimens with a higher value for BED1.7 were associated with five cases of cord myelopathy, if the spinal cord was not shielded. In poor performance status patients the model suggested that the optimal regimen was a single fraction of 10 Gy because this resulted in an equivalent degree of symptom control as other regimens, needed only one hospital visit and was less likely to result in cord damage, thus, allowing for the possibility of retreatment at a later date.


British Journal of Cancer (1998) 78(12), 1629–1633
© 1998 Cancer Research Campaign

### Radiobiological prediction of normal tissue toxicities and tumour response in the radiotherapy of advanced non-small-cell lung cancer

**JM Singer[1], P Price[2] and RG Dale[3]**

[1]Department of Clinical Oncology, Hammersmith Hospital, Du Cane Road, London W12 0HS, UK; [2]Section of Cancer Therapeutics, Division of Medicine, Imperial College School of Medicine, Hammersmith Campus, London W12 0NN, UK; [3]Department of Radiation Physics and Radiobiology, Hammersmith Hospitals NHS Trust, Charing Cross Hospital, Fulham Palace Road, London W6 8RF, UK

**Summary** A number of randomized studies have been carried out in the UK and USA to determine the optimal radiotherapy dose schedule for advanced non-small-cell lung cancer (NSCLC). We have examined eight radiotherapy regimens from data taken from four randomized phase III studies carried out in the UK (1264 patients): 10 Gy single fraction; 17 Gy in two fractions over 8 days; 30 Gy in ten fractions over 14 days; 22.5 Gy in five fractions in 5 days; 27 Gy in six fractions over 11 days; 30 Gy in six fractions over 11 days; 36 Gy in 12 fractions over 16 days; and 39 Gy in 13 fractions over 17 days. We compared the clinical results in palliation, toxicity and survival with four regimens taken from one randomized study from the USA (365 patients): 40 Gy in 20 fractions over 4 weeks; 40 Gy 'split course' in ten fractions in 4 weeks; 50 Gy in 25 fractions over 5 weeks; and 60 Gy in 30 fractions over 6 weeks. Using the linear-quadratic (LQ) radiobiological model, we have calculated the radiobiological equivalent dose (BED) for acute-reacting tissues ($BED_{10}$), late-reacting tissues ($BED_{1.7}$) and tumour ($BED_{25}$), and related the predicted response to the observed response in each tissue. There was a good correlation between the predicted response and the reported response in the case of late-reacting tissue toxicity and tumour response. The model confirmed that, in good performance status patients, a higher value for $BED_{25}$ correlated with a higher degree of local control and survival and that radiotherapy regimens with a higher value for $BED_{1.7}$ were associated with five cases of cord myelopathy, if the spinal cord was not shielded. In poor performance status patients the model suggested that the optimal regimen was a single fraction of 10 Gy because this resulted in an equivalent degree of symptom control as other regimens, needed only one hospital visit and was less likely to result in cord damage, thus, allowing for the possibility of retreatment at a later date.

**Keywords:** radiobiological modelling; non-small-cell lung cancer; radiotherapy

One consistency in the use of radiotherapy for non-small-cell lung cancer (NSCLC) is the continued variation in the radiation dose and schedule. The great majority of patients with NSCLC are incurable at the time of diagnosis, yet there is disagreement among radiation oncologists as to whether such patients should receive higher doses of radiotherapy in an attempt to gain a greater degree of local control. Higher doses of radiotherapy may be given at the cost of time and an increase in normal tissue toxicity. Moreover, it is not clear whether improved thoracic control leads to any further benefit in palliation or improvement in survival in patients with locally advanced or metastatic disease.

In this respect, there appears to be a divide in radiation treatment strategies between North America and the UK. In North America, radiation doses are typically higher than in the UK and are fractionated over 4–6 weeks. Perez et al (1980) carried out a study in which 365 patients with locally advanced NSCLC were randomized to receive:

1. a 'split' course of 20 Gy in five fractions, a 2-week break, 20 Gy in five fractions;
2. 40 Gy in 20 fractions, over 4 weeks;
3. 50 Gy in 25 fractions over 5 weeks;
4. 60 Gy in 30 fractions over 6 weeks.

A significant degree of intrathoracic tumour control was seen with increasing radiation dose and this correlated with a modest improvement in survival (37–47 weeks). The split course produced the worst results. Significant poor prognostic indicators were found to be weight loss of > 10%, age > 70 years and low Karnofsky score.

In contrast, the Medical Research Council Lung Cancer Working Party (MRC LCWP) and the Bristol group sought to explore the relationship between radiation dose schedule and symptom control, thus the primary end point has been a measurement of palliation rather than survival. Four randomized studies have been carried out:

1. 30 Gy in ten fractions over 2 weeks vs. 17 Gy in two fractions in 8 days, in patients with locally advanced or metastatic NSCLC (all performance statuses included) (MRC LCWP, 1991);
2. 17 Gy in two fractions over 8 days vs. a single 10 Gy, in poor performance patients with locally advanced or metastatic NSCLC (MRC LCWP, 1992);
3. 17 Gy in two fractions over 8 days vs. 22.5 Gy in five fractions over 5 days, in locally advanced or metastatic disease (all performance statuses included) (Rees et al 1997);

*Received 23 October 1997*
*Revised 2 March 1998*
*Accepted 29 April 1998*

*Correspondence to:* RG Dale

4. 39 Gy in 13 fractions over 17 days (or 36 Gy in 12 fractions over 16 days) vs. 17 Gy in two fractions, in good-performance patients with locally advanced or metastatic NSCLC (MRC LCWP, 1996).

In a fifth report on radiation myelopathy, using MRC Lung Cancer Working Party data, an additional two regimens are mentioned: 30 Gy in six fractions over 11 days and 27 Gy in six fractions over 11 days (Macbeth et al, 1996). These schedules are shown in Table 1, but full data on degree of palliation and survival are not available in the published literature.

The conclusions from the first three studies indicated that 30 Gy in ten fractions, 17 Gy in two fractions, 22.5 in five fractions or a single fraction of 10 Gy were equivalent in terms of symptom control, and there was no significant difference in overall survival. However, the fourth study revealed that there was a modest improvement in survival, in the good-performance group, for those treated with the 39 Gy in 13 fraction arm (or 36 Gy in 12 fractions over 16 days) (9 months vs. 7 months), but at a cost of normal tissue toxicity and inferior percentages for palliation.

Because these patients were treated with radiation only, publications such as these enable us to use such data for dose–response studies. Currently, many groups are using radiation in conjunction with chemotherapy, or chemotherapy only, thus further pure radiation studies appear unlikely. These studies provide an opportunity to examine the relationship between radiation dose and response. The purpose of this study was to compare the biological equivalent dose (BED) to three main tissues: acute-reacting tissues, late-reacting tissues and tumour, of each radiotherapy regimen (see Tables 1 and 2). We aimed to determine which radiotherapy regimen was likely to cause the least morbidity for the equivalent or superior anti-tumour effect.

### METHODS

Published data from the four UK NSCLC trials and the trial by Perez et al (1980) were analysed (see Tables 1 and 2).

BED was calculated using the linear quadratic (LQ) model (Barendsen, 1982; Fowler, 1989). The model was used in its simplest form, i.e. the BED for each tissue was calculated using equation (1) from knowledge of the number of fractions ($N$), dose per fraction ($d$) and tissue $\alpha/\beta$ ratio:

$$BED = Nd\,[1 + d/(\alpha/\beta)] \qquad (1)$$

Alternative forms of the model allow for the effect of concurrent repopulation in tumours or acute-responding tissues, but these effects were not considered here as there is a paucity of data relating to such events. Although there is fairly extensive data relating to repopulation rates of squamous cell carcinoma of the head and neck (Hendry et al, 1996), there is little reliable data relating to repopulation of NSCLC or acute-responding tissues. Furthermore, it is not clear how palliation response is influenced (if at all) by repopulation. Corrections for repopulation would have the effect of reducing the calculated BED values for acute and tumour responses, but the amount of the reduction is unlikely to be significant for the shorter treatment regimens, i.e. as used in the MRC studies.

The $\alpha/\beta$ values reflect the fractionation sensitivity of specific radiation responses and, for acute-reacting tissue, spinal cord and tumour (NSCLC), were respectively assumed to be 10, 1.7 and 25 Gy. The choice of $\alpha/\beta$ = 10 Gy to characterize the acute responses is selected with reference to data compiled by Joiner and van der Kogel (1997), and which show a moderately narrow spread of values (typically 7–13 Gy) for a range of early reactions. The selection of a low $\alpha/\beta$ value of around 1.7 Gy for the spinal cord is important when considering potential long-term damage to this structure. Joiner and van der Kogel (1997) have tabulated experimentally determined values of this parameter and it is clear that it is likely to be significantly lower than the generic value of 3 Gy, which is often used for quantifying late reactions. This point is amplified in the Results section. For the tumour response, an $\alpha/\beta$ value of 25 Gy has been assumed. This implies a low sensitivity to changes in fractionation, but the value selected is not especially critical in the context of this paper. Assuming the more common generic value of 10 Gy does not change any of the conclusions. Similarly, the relative predictions based on BED calculations are unchanged when tumour $\alpha/\beta$ is increased from 25 Gy to much larger values. Throughout this article, each calculated BED value is followed by a suffix which identifies the assumed $\alpha/\beta$ value.

The incidence of dysphagia has been assumed to be an indicator of acute-reacting tissue toxicity, and the incidence of spinal cord toxicity has been assumed to be a measurement of late tissue damage.

Accurate measurement of tumour response is problematic because this is not directly measured in any study. Overall survival may be considered as an indicator of likely intrathoracic tumour response, but this has been disputed and some consider survival dependent more on the presence of distant metastases. Therefore, we have also considered the degree of palliation of cough, haemoptysis and chest pain as an indicator of tumour response.

### RESULTS

### UK data

#### *Spinal cord response*

In Table 1, the eight radiotherapy regimens are listed in descending order of magnitude of their spinal cord toxicity, as judged by the rankings of the values of $BED_{1.7}$. Also shown in the table is the number of patients in each group.

Of the seven regimens, the first five are associated with nominal cord BEDs of 98 Gy or more. Five cases of myelopathy occurred in the starred regimens, which happen also to involve the largest number of patients. On the basis that a $BED_{1.7}$ of ~ 100 Gy carries some risk of late cord damage, three other regimens may fall into this category: 6 × 5 Gy, 12 × 3 Gy and 6 × 4.5 Gy. The lack of any observed cases may be due to the smaller numbers of patients involved in these three groups.

The bracketed numbers next to each value of $BED_{1.7}$ are the alternative ranking orders obtained if an $\alpha/\beta$ value of 3 Gy is used in place of 1.7 Gy. In the absence of specific values for $\alpha/\beta$, a generic value of 3 Gy is often used to characterize late-reacting responses. It will be noted that the ranking changes are relatively small, but that the largest (a drop from third to fifth rank) is seen with 2 × 8.5 Gy, which is one of the regimens with which cord myelopathy has been reported. This supports the premise that a value of $\alpha/\beta$ lower than 3 Gy should always be used in assessing cord response because the enhanced sensitivity to small changes in dose per fraction is then better predicted.

#### *Acute tissue response*

Acute toxicity appears to rise fairly quickly for a $BED_{10}$ in excess of 30 Gy: reported dysphagia was significantly worse in the 39 Gy

© Cancer Research Campaign 1998

**Table 1** Radiotherapy dose fractionation and biologically equivalent dose (BED) in three tissues: acute-reacting tissue ($BED_{10}$), spinal cord ($BED_{1.7}$) and tumour ($BED_{25}$). The regimens are listed in order of decreasing spinal cord toxicity as determined from the $BED_{1.7}$ values. The bracketed numbers in the $BED_{1.7}$ column show how the ranking order is changed if a generic α/β of 3 Gy is used instead

| Fractionation | No. of patients | $BED_{10}$ (Gy) | $BED_{1.7}$ (Gy) | $BED_{25}$ (Gy) |
|---|---|---|---|---|
| 6 × 5 Gy in 11 days | 36 | 45 | 118 (1) | 36 |
| 13 × 3 Gy in 16 days[a,b] | 153 | 51 | 108 (2) | 44 |
| 2 × 8.5 Gy in 6 days[a] | 635 | 31 | 102 (5) | 23 |
| 12 × 3 Gy in 15 days[b] | 86 | 47 | 100 (3) | 40 |
| 6 × 4.5 Gy in 7 days | 47 | 39 | 98 (4) | 32 |
| 10 × 3 Gy in 13 days | 88 | 39 | 83 (6) | 34 |
| 5 × 4.5 Gy in 4 days | 105 | 33 | 82 (7) | 27 |
| 1 × 10 Gy in 0 days | 114 | 20 | 69 (8) | 14 |

[a]Cord myelopathy reported; [b]significantly worse dysphagia reported.

**Table 2** Radiotherapy dose fractionation and biological equivalent dose (BED) in three tissues: acute-reacting tissue ($BED_{10}$), spinal cord ($BED_{1.7}$) and tumour ($BED_{25}$). Data taken from NSCLC study (Perez et al, 1980)

| Fractionation | No. of patients | $BED_{10}$ (Gy) | $BED_{1.7}$ (Gy)[a] | $BED_{25}$ (Gy) | Incidence intrathoracic recurrence (%) |
|---|---|---|---|---|---|
| 30 × 2 Gy in 39 days | 84 | 72 | 131 | 65 | 33 |
| 25 × 2 Gy in 32 days | 91 | 60 | 109 | 54 | 39 |
| 20 × 2 Gy in 25 days | 97 | 48 | 87 | 43 | 49 |
| 'Split course': 5 × 4 Gy, 2-week break, 5 × 4 Gy (overall: 25 days) | 93 | 56 | 134 | 46 | 44 |

[a]In this study, lead shielding was used in the posterior portals to shield the spinal cord. Therefore, the dose to the spinal cord is uncertain. Moreover, the incidence of cord myelopathy is not reported.

in 13 fractions ($BED_{10}$ = 51 Gy) or 36 Gy in 12 fractions ($BED_{10}$ = 47 Gy) compared with 17 Gy in two fractions ($BED_{10}$ = 31 Gy) (MRC LCWP, 1996). In addition, a single fraction of 10 Gy ($BED_{10}$ = 20 Gy) produced very little dysphagia compared with 17 Gy in two fractions (23% versus 56%) (MRC LCWP, 1992). Macbeth et al (1996) have shown how the per cent of time for which dysphagia was reported may be fitted with a radiobiological model that combines radiation-induced stem cell depletion with subsequent stem cell repopulation. Although dysphagia is transient, its onset is most likely to be governed by the initial net cell depletion, of which a straightforward $BED_{10}$ value may provide a reasonably adequate measure for the purpose of ranking likely incidence of dysphagia.

### *Tumour response*

As discussed above, measurement of tumour response was indirect. $BED_{25}$ is highest for 39 Gy in 13 fractions and 36 Gy in 12 fractions. It is interesting to note that these regimens produced the longest survival times, yet were inferior with regard to palliation of symptoms. Therefore, no firm conclusions can be drawn with regard to the radiobiological model predicting tumour response in these studies. Although these BEDs are uncorrected for possible repopulation, it is easy to show that, even if repopulation in NSCLC occurred at the high rate observed in head and neck tumours, the rankings of the two most potent regimens would not be altered.

### USA data (Perez et al, 1980)

The four regimens are listed in Table 2. It is clear from the modelling that 60 Gy in 30 fractions produces the highest likely tumour response ($BED_{25}$) and highest acute-reacting tissue response ($BED_{10}$). The split course regimen produced the highest late tissue response for a relatively low tumour response. However, in this study, partial shielding of the spinal cord was used and accurate data on the dose received at cord level is not available. Also of note is that the predicted tumour response ($BED_{25}$) corresponds very well to the data for the incidence of intrathoracic control and overall survival (see Table 2). Twenty gray in ten continuous fractions ($BED_{25}$ = 43.2 Gy) has the highest incidence of intrathoracic recurrence (49%), and 60 Gy in 30 fractions ($BED_{25}$ = 64.8 Gy) had the lowest (33%).

### DISCUSSION

Radiobiological modelling may have a place as a complement to clinical judgement, as, for example, in cases in which alternative treatments for a particular site show unexpected differences in radiation responses (Dale et al, 1997). The UK NSCLC studies and the study by Perez et al (1980) provide useful data with which to test the ability of radiobiological models to predict ranking orders of likely response. It is probable that further data on radiation dose–response will no longer be available, as many groups are

© Cancer Research Campaign 1998

now using radiotherapy in conjunction with chemotherapy. Because the UK studies are prospectively carried out primarily to measure symptom control, data collection for acute and late tissue toxicity was better than the measurement of tumour response. In contrast, a more aggressive approach is adopted by Perez et al (1980), in that relatively higher doses of radiation are given, with the primary aim of gaining intrathoracic control. Thus, data are more complete on tumour response and less detailed on symptom control or toxicity.

It is still unclear from this work and the literature as to whether we can accurately predict the best regimen for tumour response, and whether this will lead to the best symptom control and survival. Using the UK data, this exercise predicts that 39 Gy in 13 fractions is the most likely to produce the greatest tumour cell kill ($BED_{25}$ = 44 Gy) although this also produced two incidences of spinal cord myelopathy, which is in keeping with the model prediction for late tissue damage ($BED_{1.7}$ = 108 Gy). Thirty-nine gray in 13 fractions produced a modest improvement in survival compared with 17 Gy in two fractions (9 months vs. 7 months), but no advantage in symptom control. The entry criteria of the study by Perez et al (1980) stipulated that patients should have had locally advanced but not metastatic NSCLC. Thus, these patients were not strictly comparable to many of the patients in the UK NSCLC studies, which allowed inclusion of patients with metastatic disease. This may explain why median survival times were overall greater in the Perez study compared with the MRC studies. However, there is evidence of a dose–response effect in that the highest radiobiological dose of 60 Gy in 30 fractions produced the best intrathoracic tumour control and this corresponded to longer survival times. Notably, the methods used to define local control were not clearly stated and this is a problem in many studies of this nature. It is presumed that recurrence was detected radiographically.

Taken together, the studies by Perez et al (1980) and the MRC Lung Cancer Working Party (1996) indicate that, in the absence of metastases, in good performance status (WHO grade 0–2), a radiation dose, which translates to a $BED_{25}$ of above 40 Gy, produces a benefit in local control and this appears to translate to a survival advantage. However, consideration must be given to the fact that the improvement in intrathoracic control and survival may be modest and at the expense of increased normal tissue toxicity and number of hospital visits.

An audit of the radiation oncologists in the UK indicated that there is a diversity in the radiation schedules used for treatment of NSCLC (Maher et al, 1993). A reason for the variability may be that radiation oncologists are aware of a possible dose–response in the good performance status patients and prefer to give their patients the best possible chance of increased survival. Until more accurate prognostic indicators are discovered, a number of clinicians may continue to use prolonged courses of radiotherapy to treat many of their patients with advanced NSCLC.

The situation for the poor performance patients is clearer: median survival times are considerably lower (3–4 months) and the aim should, therefore, be relief of symptoms with the least toxicity. Data from the MRC studies indicate that a single fraction of 10 Gy is associated with the lowest BEDs for all three biological end points considered. Therefore, in a clinical situation in which a patient may be of low performance status (WHO grade 2–4), and with locally advanced or metastatic disease, a single fraction of 10 Gy produces rapid relief of symptoms at the cost of only one visit. In addition, the LQ model predicts that, of all the regimens considered, it is least likely to lead to spinal cord myelopathy and acute toxicity. There is now substantial experimental evidence that long-term recovery does occur in the irradiated cord and that retreatment (albeit with a reduced dose) is feasible (Stewart, 1997). Should the patient survive longer than expected, the $1 \times 10$ Gy regimen does allow some potential for retreatment at a later date if new symptoms arise, although in individual cases the true (rather than nominal) dose to the cord would need to be taken into account.

The UK lung trials clearly indicate that the considerations which apply when identifying effective palliative treatments in radiotherapy are quite different from those which apply to radical treatments. On the basis that palliative treatments are not usually expected to involve near-tolerance irradiation of normal structures, it might appear that there is little scope for biological modelling predictions in this area. However, as this intercomparison shows, even simple modelling goes some way to providing supporting information regarding the likely expected ranking of various regimens, and prospective use of such modelling may help focus on the issues involved and help with the early rejection of regimens which are unlikely to achieve the clinically desired aim.

### CONCLUSION

The linear quadratic model predicted the likely responses of acute- and late-normal effects associated with the treatment of NSCLC with reasonable consistency. The same appears to be true of the tumour responses, but this is a more problematic area as the role of modelling in quantifying tumour palliation has yet to be fully identified. We have used the model to compare various radiotherapy regimens used in the UK and the USA. The model predicts that in good performance status, non-metastatic NSCLC patients the higher radiation dose schedules (39 Gy in 13 fractions, 50 Gy in 25 fractions, 60 Gy in 30 fractions) are associated with greater tumour cell kill, which has been shown to translate to a modest improvement in local control and survival but no improvement in palliation. In particular, the 'split course' was predicted to have a poorer tumour response and, because of the rather large dose per fraction involved, has the highest likelihood of spinal cord toxicity.

In the situation of poor performance status (WHO grade 2–4), the linear quadratic model predicts that a single fraction of 10 Gy is the optimum regimen for palliation and leaves the possibility for future radiotherapy if necessary.

### REFERENCES

Barendsen GW (1982) Dose fractionation, dose-rate and iso-effect relationships for normal tissue responses. *Int J Radiat Oncol Biol Phys* **8**: 1991–1997

Dale RG, Jones B and Price P (1997) Inadequacy of iridium implant as sole radiation treatment for operable breast cancer. *Eur J Cancer* **33**: 1707–1708

Fowler JF (1989) The linear-quadratic formula and progress in fractionation. *Br J Radiol* **62**: 679–694

Hendry JH, Bentzen SM, Dale RG, Fowler JF, Wheldon TE, Jones B, Munro AJ, Slevin NJ and Robertson AG (1996) A modelled comparison of the effects of using different ways to compensate for missed treatment days in radiotherapy. *Clin Oncol* **8**: 297–307

Joiner MC and van der Kogel AJ (1997) The linear-quadratic approach to fractionation and calculation of isoeffect relationships. In *Basic Clinical Radiobiology*, 2nd edn, Steel GG (ed.), pp. 106–122. London: Edward Arnold

Macbeth FR, Wheldon TE, Girling DJ, Stephens RJ, Machin D, Bleehan NM, Lamont A, Radstone DJ and Reed NS (1996) Radiation myelopathy: estimates of risk in 1048 patients in three randomised trials of palliative radiotherapy for non-small cell lung cancer. *Clin Oncol* **8**: 176–181

© Cancer Research Campaign 1998

Maher EJ, Timothy A and Squire CJ (1993) Audit: the use of radiotherapy for NSCLC in the UK. *Clin Oncol* **5**: 72–79

MRC LCWP (Medical Research Council Lung Cancer Working Party) (1991) Inoperable non-small-cell lung cancer (NSCLC): a Medical Research Council randomised trial of palliative radiotherapy with two fractions or ten fractions. *Br J Cancer* **63**: 265–270

MRC LCWP (Medical Research Council Lung Cancer Working Party) (1992) A Medical Research Council (MRC) randomised trial of palliative radiotherapy with two fractions or a single fraction in patients with inoperable non-small-cell lung cancer (NSCLC) and poor performance status. *Br J Cancer* **65**: 934–941

MRC LCWP (Medical Research Council Lung Cancer Working Party) (1996) Randomised trial of palliative two-fraction versus more intensive 13-fraction radiotherapy for patients with inoperable non-small cell lung cancer and good performance status. *Clin Oncol* **8**: 167–175

Perez CA, Stanley K, Ruben P, Karma S, Bride L, Perez-Tame R, Brown GS, Concannon J, Rotman M and Seydel HG (1980) A prospective randomised study of various irradiation doses and fractionation schedules in the treatment of inoperable non-oat-cell carcinoma of the lung. *Cancer* **45**: 2744–2753

Rees GJG, Devrell CE, Barley VL and Newman HFV (1997) Palliative radiotherapy for lung cancer: two versus five fractions. *Clin Oncol* **9**: 90–95

Stewart FA (1997) Re-treatment tolerance of normal tissues. In *Basic Clinical Radiobiology*, 2nd edn. Steel GG (ed.), pp. 203–211. London: Edward Arnold